# Incidence of spinal cord injuries in Germany

**DOI:** 10.1007/s00586-022-07451-0

**Published:** 2022-11-13

**Authors:** Yannick Rau, Arndt-Peter Schulz, Roland Thietje, Ludwig Matrisch, Jasper Frese, Sven Hirschfeld

**Affiliations:** 1 Faculty of Medicine, Universität zu Lübeck, Lübeck, Germany; 2 Spinal-Cord-Injury Centre, BG Klinikum Hamburg, Hamburg, Germany; 3 Department of Trauma, Orthopaedics and Sports Surgery, BG Klinikum Hamburg, Hamburg, Germany

**Keywords:** Spinal cord injury, Incidence, Epidemiology, Germany

## Abstract

**Purpose:**

The goal of this study was to provide recent data on incidence of spinal cord injuries (SCI) in Germany.

**Methods:**

The source of information was data collected via the mandatory submission of ICD-10 GM Codes by German public hospitals after patient discharge. Data from 2013 to 2020 were retrieved from the databases of the Federal Bureau of Statistics. ICD-10 Codes for acute SCI were identified. Statistical analysis was performed using Jamovi and Excel.

**Results:**

A total of 10,360 patients were reported, of whom 58.7% suffered from a cervical, 30.8% a thoracic and 10.4% a lumbar lesion. Two peaks in incidence were observed at approximately 30 and 70 years old. A population-size-adjusted overall incidence of 15.73 (SD 0.77) per million per year was calculated. We calculated the incidences in several subpopulations and discovered significantly higher incidences among males and among those over the age of 60. We discovered that differences in age groups mainly concerned injuries of the upper spine, with the incidence in the lumbar spine being similar among age groups. In addition, we found that while the probability of suffering from SCI increases with age, the relative risk of suffering from a complete injury decreases.

**Conclusions:**

This study closes a long-lasting gap in epidemiological data regarding SCI in Germany, specifically by updating the incidence rates. We found that incidence depends on age, gender and type of lesion. We also provide some new angles for future research, especially considering the relative reduction in complete injuries among the elderly.

## Introduction

Spinal cord injuries (SCI) are a worldwide phenomenon that were even mentioned in the scriptures of ancient Egypt [1]. Although significant improvements in acute care, specialized rehabilitation and prevention have been made since then, life expectancy for SCI victims in Germany remains limited and no cure has yet been found [2–4]. This underlines the importance of preventative measures and further research into all aspects of SCI and a constant necessity for re-evaluation of the current situation. This requires reliable epidemiological data at a local level, since cultural, economic and sociological aspects may differ drastically from country to country and therefore have a different influence on risks and benefits when it comes to SCI.

International data suggest an incidence of between 3.6 up and 195.4 per million [5]. More developed countries tend to have a lower incidence rate than their undeveloped counterparts [6]. However some studies have suggested that reported numbers may be unreliable, in particular in undeveloped countries, as national registries or databases are missing [7].

Unfortunately, there is a similar problem in Germany as there are very few epidemiological studies on SCI because there are no national clinical databases that could be used as a basis for such studies. The most reliable sources of data are studies that are using big but not all-encompassing databases like the TraumaRegister DGU. Such a study was performed by Stephan et al. in 2015 using data from 2002 to 2012 [8]. And while this study provides clinical details on patients with spinal cord injuries, it only allows for an indirect approach to incidence which again leads to an approximation at best, as it cannot be expected to include all SCI patients. An older study published in 2004 by Exner provided data from all SCI specialized centres in Germany from 1976 to 2003. An average of 1780 new admissions per year were reported [9]. And although very commendable for its long research period as well as the wide range of hospitals included, incidences may have changed within the last 20 years. The scarcity of information remains unsatisfactory. This study aimed to change the uncertainty when it comes to the basic incidence and age distribution of traumatic spinal cord injuries.

## Methods

### Data collection

Data were retrieved from the German Federal Bureau of Statistics (Statistisches Bundesamt), Department of the Interior. The Federal Bureau of Statistics collects the ICD-10 GM and OPS Codes of all patients admitted to a German hospital every year and is therefore able to provide detailed information on how many patients were treated as a result of distinct illnesses or injuries [10].

Datasets from 2013 to 2020 of all patients released from hospitals with an S-code diagnosis, corresponding to the WHO group XIX (Injury, poisoning and certain other consequences of external causes) were provided and specific injury types were identified [11]. In particular, injuries to the cervical, thoracic and lumbar spinal cord were identified and extracted.

The ICD-10 GM codes S14.10 to .13 for cervical injuries, S24.10 to .12 for thoracic injuries and S34.10 to.11 and S34.18 for lumbar injuries were identified as targets of interest.

S14.11, S24.11 and S34.10 are complete or AIS type A lesions. S14.10, S24.10 and S35.18 are considered to be SCI of interest but are not considered to be potentially complete (ASIA Impairment Scale type A) lesions.

### Statistical analysis

Jamovi (Version 2.3.11, The Jamovi Project, Sydney, Australia) and Excel (Version 2205, Microsoft, Redmond, USA) were used to perform the statistical analysis.

Incidences were calculated using population data also provided by the German Federal Bureau of Statistics for each year of analysis.

The study population was divided into two age groups: those above the age of 60 and those below. Although this division was arbitrary, it was established in recent literature as a common cut-off to divide the population into a potential workforce and those who are close to or in retirement [4].

Statistical comparison between values was performed using Student’s t test. A difference was estimated to be statistically significant at *p* < 0.05. Effect size is stated as Cohen’s *d*. Cohen’s *d* > 0.8 is considered to represent a large effect [12].

Incidence rate is always stated as per million and per year.

## Results

A total of 10,360 spinal cord injuries were reported, which translates to a mean of 1295 (SD 63.34) new SCI each year. Of these, 7746 patients (74.8%) were male. A total of 6086 patients (58.7%) suffered from a cervical lesion, 3194 (30.8%) from a thoracic lesion and 1080 (10.4%) from a lumbar lesion.

**Fig. 1:**
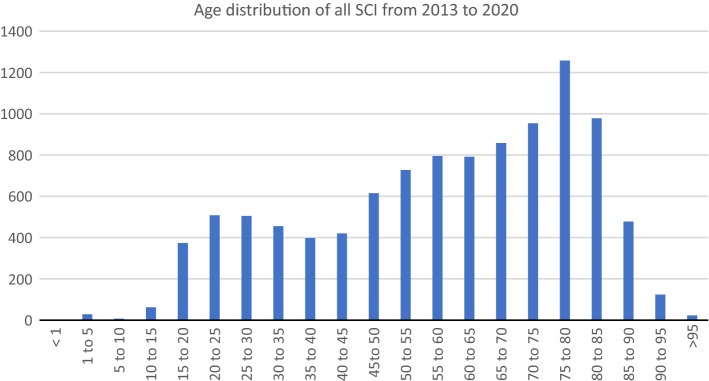
Age distribution of absolute SCI occurrence in 5-year intervals

The age distribution is shown in Fig. 1. Two peaks are apparent—one between 20 and 30 years old and one after the age of 70; 5,464 of all patients (52.7%) were over the age of 60.

The absolute number of new SCI each year without adjustment for population size is shown in Table 1.

**Table 1:** New SCI each year

	All new SCI	< 60 years old	> 60 years old
2013	1210	610	600
2014	1333	608	725
2015	1321	600	721
2016	1374	663	711
2017	1325	605	720
2018	1294	641	653
2019	1314	599	715
2020	1189	570	619

**Fig. 2:**
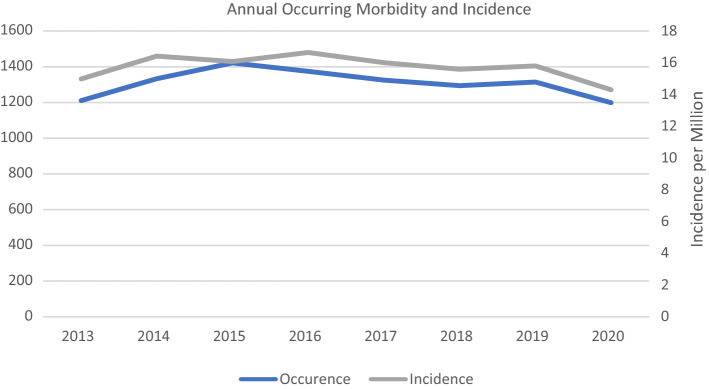
Annual morbidity and incidence of SCI per million in Germany

Adjusted for annual population size in each year, the mean total incidence was 15.73 (SD 0.77) per million. Incidences varied slightly and have declined in recent years while overall occurrence remained steady during the analysed period (Fig. 2).

The total incidence of SCI within the male population at a mean of 23.88 (SD 1.50) per million was significantly higher than in the general population throughout the analysed period (*p* < 0.001).

**Fig. 3:**
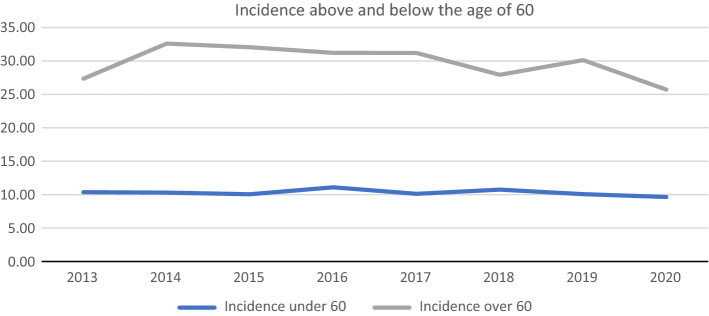
Incidence per million above and below the age of 60

Incidence rates above and below the age of 60 were also calculated (Fig. 3).

While the incidence in those below the age of 60 remained roughly the same at a mean of 10.31 (SD 0.45) per million with a very limited range, the incidence in those above the age of 60 has dropped relatively steadily since 2014 from 27.34 to 25.7 in 2020. The mean incidence was 29.77 (SD 2.48) per million, which again differed significantly (*p* < 0.001) from the incidence in those under the age of 60.

At the same time however, population size is increasing with the main body of growth being made up by those over the age of 60 (Table 2). With an increase in population size of almost 10% in that group alone, the reduction in incidence of only about 4.5% was essentially nullified. Considering the overall incidence only dropped from 14.98 to 14.30 in the observed period, while population size increased by about 3.0%, a decrease in absolute SCI occurrence due to reduced incidence rates is unlikely and was not observed.

**Table 2:** Changes in population size above and below the age of 60

	Population size	< 60 years old	> 60 years old
2013	80,767,463	58,819,703	21,947,760
2014	81,197,537	58,955,849	22,241,688
2015	82,175,684	59,673,450	22,502,234
2016	82,521,653	59,730,668	22,790,985
2017	82,792,351	59,707,587	23,084,764
2018	83,019,213	59,641,767	23,377,446
2019	83,166,711	59,428,258	23,738,453
2020	83,155,031	59,065,258	24,089,773

**Fig. 4:**
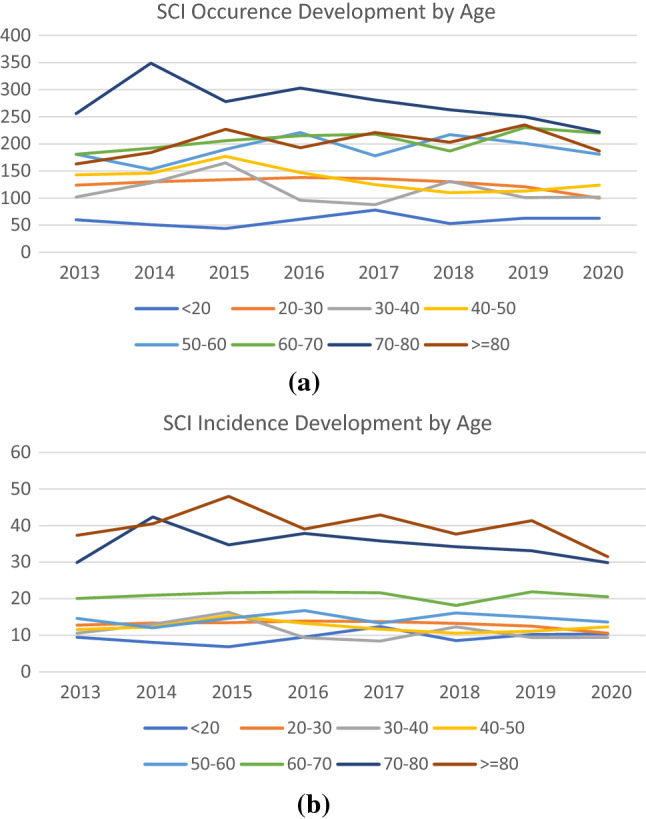
**a** Change in SCI occurrence by year and age. **b** Change in SCI incidence per million by year and age

The change in absolute occurrence and incidence in all age groups can be seen in Fig. 4a, b. Only the group comprising those between the age of 70–80 showed a relevant decrease over the observed period, while the incidence in those between the age of 60 to 70 seemingly increased. Considering the sideways trend of incidences in all age groups, this may be due to ongoing changes within the demographic structure, as some generations showed a higher birth rate than others.

The mean incidence of complete (ASIA Impairment Scale type A) SCI in the two age groups was also calculated and a *t* test was performed [13]. The incidence of complete SCI was 5.08 (SD 0.65) per million and differed significantly from the overall SCI incidence (*p* < 0.001). Additional incidence rates for each spinal region and a general overview are presented in Table 3.

**Table 3:** Incidences per million per year (SD = Standard Deviation)

	Mean	Median	SD	Minimum	Maximum
Incidence of all SCI	15.727	15.902	0.770	14.299	16.650
Incidence of complete SCI	5.180	5.059	0.659	4.353	6.240
Incidence over 60	29.765	30.655	2.484	25.700	32.600
Incidence under 60	10.305	10.220	0.449	9.650	11.100
Incidence male	23.877	723.762	1.502	21.492	26.595
Incidence female	7.535	8.646	2.271	3.810	9.309
Incidence cervical	9.238	9.352	0.626	8.262	10.058
Incidence thoracic	4.849	4.926	0.211	4.413	5.038
Incidence lumbar	1.639	1.651	0.185	1.241	1.823
Incidence cervical under 60	4.942	4.937	0.378	4.405	5.609
Incidence cervical over 60	20.362	21.433	1.971	17.061	22.049
Incidence thoracic under 60	3.738	3.822	0.262	3.352	4.120
Incidence thoracic over 60	7.727	7.596	0.634	7.015	8.992
Incidence lumbar under 60	1.626	1.646	0.263	1.022	1.878
Incidence cervical complete	2.326	2.391	0.275	1.958	2.611
Incidence thoracic complete	2.368	2.441	0.264	2.012	2.623
Incidence lumbar complete	0.540	0.305	0.488	0.195	1.407
Incidence cervical complete	2.326	2.391	0.275	1.958	2.611
Incidence complete under 60	3.851	3.845	0.333	3.471	4.556
Incidence complete > 60	7.610	7.421	0.968	6.517	9.307

The biggest differences from the mean SCI incidence were seen in males and in those aged over 60 years old.

A particularly big impact of age was apparent in cervical SCI as those who were over the age of 60 were about four times more likely to suffer from cervical SCI than those below the age of 60.

Patients of all ages were significantly less likely to suffer from a complete lumbar SCI, and while there was a twofold higher risk of suffering from a cervical lesion than from a thoracic lesion, there was no significant increase in risk of suffering from a complete cervical lesion than from a complete thoracic lesion (*p* = 0.363).

At the same time, those who were over 60 years old were only two times as likely to suffer from complete thoracic SCI, while the incidence of complete lumbar SCI did not differ between age groups (*p* = 0.41).

The incidence of complete SCI in those over the age of 60 was almost double that in those below the age of 60. However, this relationship did not reflect the overall ratio of SCI, which occurred almost three times as often over the age of 60. Incomplete injuries are therefore more uncommon among Germans under the age of 60, when adjusted for overall incidence.

Table 4 shows the results of one-sample *t* tests of some incidences against overall SCI incidence. The incidence in males and those over and under the age of 60 was shown to differ significantly (*p* < 0.05) from the overall mean SCI incidence with a large effect size of over 0.8 in all cases.

**Table 4:** One-sample t test against mean overall SCI incidence

	*p*	Effect size
Incidence male	< 0.01	4.728
Incidence under 60	< 0.01	− 12.083
Incidence over 60	< 0.01	5.651

*H*_a_ *μ* ≠ 15.727

In addition, incidences in those groups under and over the age of 60 were shown to differ significantly from each other for all types of SCI except lumbar SCI, where there was no significant difference between those under and over the age of 60 years old (*p* = 0.612). The incidence of lumbar SCI therefore does not appear to depend upon age.

## Discussion

Knowledge about the incidence and distribution of SCI among the population as well as potential differences in risks of different injury types could lead to a new approach in prevention and aftercare. Reliable epidemiological data are the groundwork for future research addressing potential risk factors, preventative measures and the economic impacts of SCI.

This study’s aim was to close a long-lasting gap in SCI research in Germany, where imprecise approximations have been used to determine SCI incidence and reliable data are scarce.

We used a different approach to achieve a more precise estimation of incidences and distribution by relying on data collected by a federal authority instead of using a common single or multicentre approach. Besides delivering a more complete picture, this approach will be easier to reproduce in future research if newer data are required.

Even though this study has delivered some long-needed metrics, it is not without its limitations.

First of all, only hospitals that are using the diagnosis-related groups (DRG) system as their means of treatment compensation are required to submit data to the federal authorities. This however does not affect the study’s meaningfulness in a relevant way as essentially all primary care facilities in Germany are using and required to use the DRG system. The hospitals are also only required to submit diagnoses upon patient discharge or death, which means that a submitted SCI may not have occurred in the same year as it was documented. However, this should not significantly influence the overall statistics, as it is a recurring phenomenon, and it should therefore only influence the precision of individual datasets for each year.

Secondly, no clinical aspects that are not implicated within the analysed ICD-10 GM code could be included in the analysis, as there is no way to connect a patient to different diagnoses.

In addition, the codes identified for analysis include diagnoses that are not defined as precisely as they would need to be to fit into certain categories. These include S14.10, S24.10 and S34.18 in particular. S14.10 and S24.10 are indeterminable lesions of the respective spinal region but are most likely incomplete lesions and sometimes those that are diagnosed by radiological diagnostics without a respective clinical manifestation. S34.18 describes “other lesions of the lumbar spinal cord” and therefore may include a variety of lesions but again is most likely an incomplete lesion without clinical significance. These three diagnoses are uncommon (about 5% of all SCI each year) and are only used if no other diagnosis can reasonably be applied because their use has negative economic implications for the respective hospital.

Fifty-nine patients (0.6% of the total study population) were reported to be 5 or less years old at the time of their SCI. Diagnosis and determination of complete or incomplete injuries relies heavily on the ASIA Impairment Scale (AIS) or the international standards for neurological classification of spinal cord injury (ISNCSCI). Current literature suggests that ISNCSCI may however not be a reliable tool to assess children under the age of 6 and therefore misdiagnoses could have affected this study’s outcome [14, 15].

Overall, this study’s results are similar to those of Stephan et al., from 2014. Males were reported to make up 72.7% of SCI victims versus 74.8% in this study; 51.8% involved the cervical spine versus 58.7% in this study; 32.4% involved the thoracic spine versus 30.8% here and 18.4% versus 10.4% involved the lumbar spine [8].

However, Stephan et al. only reported 4,285 patients suffering from SCI within the period from 2002 to 2012. Considering this study’s findings and earlier incidence reports like those from Exner in 2004 suggests a far higher incidence than can be calculated from the TraumaRegister DGU data and shows the limitations of epidemiological studies that rely on limited registries [8, 9].

We also found that while Stephan et al. reported around 65.4% of cervical lesions to be complete SCI, only about 25.6% of the cervical lesions in our study were compete injuries. Differences in thoracic and lumbar lesions were less prominent [8]. This suggests either an overreporting of complete cervical lesions or more likely an underreporting of incomplete cervical lesions to the Trauma Register DGU. This discrepancy might best be addressed and further investigated in a future cohort study conducted in a specialized care centre.

As shown herein, certain groups within the population seem to be at a particular risk of SCI with significant deviations from the overall incidence within these groups (Table 3). Male sex as well as age were discovered to correlate positively with SCI incidence. These modalities have been independently reported as potential risk factors within the international literature. The age distribution with two peaks as shown in Fig. 1 is also a common finding among international studies [6, 16–18]. The cause of SCI in both age peaks is most likely different. However, the federal bureau of statistics does not differentiate between different causes of SCI. We therefore can only base our assumptions on data published by other working groups. As research regarding epidemiological characteristics of SCI patients, especially considering causes of injury in relation to age, is rare in Germany, we use data from neighbouring countries in Europe with similar standards of living, demographics and health care systems. Smith et al. found that in Ireland people who suffer from traumatic SCI because of falls are significantly older and in-line with our discovered age peak [18]. Jonviea et al. discovered that in Switzerland younger people between 16 and 30 years old suffer more often from traumatic SCI because of sports, leisure activity or transport related accidents. Whereas low-level falls were the main reason for people above the age of 76 years to suffer from SCI [19]. We conclude that the reason for SCI in both age peaks differs because of lifestyle and comorbidities. While younger people are taking a more active role in society and are more risk taking, older people are most likely mainly at risk because of deteriorating physical capabilities, e.g. bone density and muscle strength. The prevention of falls is therefore of considerable importance.

Interestingly, we found that while the overall incidence of SCI and complete SCI increased with age, the relative probability of suffering from a complete injury decreased from 37.4% under the age of 60 to 25.6% over the age of 60. This may be due to different injury mechanisms within the two age groups but requires further investigation in future research.

Incidence and occurrence showed a sideward trend up until 2019. A drop from 2019 to 2020 could be observed. Similar findings were made by Maleitzke et al. with regard to overall orthopaedic trauma injury incidence in a level 1 trauma centre [20]. These findings were directly related to the COVID-19 shutdown in Germany. We therefore conclude that our observed drop is most likely also related to the pandemic and its consequences. The reduction in workplace related injuries, motor vehicle accidents and overall decrease in leisure activities are the most likely causes for the decline in severe spinal injuries. Further research into the specific reasons behind the decrease in spinal cord injuries during the pandemic could help identify factors suitable for preventative measures in the future.

Overall, the sideways trend of SCI incidence within the observed period in combination with an increasing population size, especially among those over the age of 60 (Table 2), indicates a continuing need for specialized care and an increase in efforts regarding SCI prevention and rehabilitation.

## Conclusions

This study was able to strengthen previous findings as well as close a long-lasting gap by delivering new data concerning the incidence of traumatic spinal cord injury in Germany.

We found that the incidence of SCI in Germany has decreased in comparison to findings from 20 years ago while remaining relatively steady over the research period. We also made some new observations on distribution in comparison to more recent studies addressing the epidemiological details of SCI patients.

## Data Availability

The datasets generated and/or analysed during the current study are available from the corresponding author on reasonable request.
